# Modeling visual-based pitch, lift and speed control strategies in hoverflies

**DOI:** 10.1371/journal.pcbi.1005894

**Published:** 2018-01-23

**Authors:** Roman Goulard, Jean-Louis Vercher, Stéphane Viollet

**Affiliations:** Aix Marseille Univ, CNRS, ISM, Marseille, France; Northeastern University, UNITED STATES

## Abstract

To avoid crashing onto the floor, a free falling fly needs to trigger its wingbeats quickly and control the orientation of its thrust accurately and swiftly to stabilize its pitch and hence its speed. Behavioural data have suggested that the vertical optic flow produced by the fall and crossing the visual field plays a key role in this anti-crash response. Free fall behavior analyses have also suggested that flying insect may not rely on graviception to stabilize their flight. Based on these two assumptions, we have developed a model which accounts for hoverflies´ position and pitch orientation recorded in 3D with a fast stereo camera during experimental free falls. Our dynamic model shows that optic flow-based control combined with closed-loop control of the pitch suffice to stabilize the flight properly. In addition, our model sheds a new light on the visual-based feedback control of fly´s pitch, lift and thrust. Since graviceptive cues are possibly not used by flying insects, the use of a vertical reference to control the pitch is discussed, based on the results obtained on a complete dynamic model of a virtual fly falling in a textured corridor. This model would provide a useful tool for understanding more clearly how insects may or not estimate their absolute attitude.

## Author summary

On the basis of vision-based feedback control of optic flow occurring during insects’ flight, we developed a dynamic model that accounts for the pitch orientation and speed in plummeting flies. We compared the hoverflies’ responses with our model and showed that an optic-flow based control strategy can be used to correct the initial pitch misorientation caused by the free fall situation. To complete the model, we combined the closed-loop control of the vertical optic flow with an additional feedback control loop based on the value of the absolute pitch orientation. The need for this measurement to stabilize the pitch orientation raises the question as whether this is also the case in dipterans. After ruling out the possibility that insects may use gravity acceleration cues to control their flight, for which no experimental evidence has been found so far, we discussed the three main sensory processes possibly involved in in their ability to control their attitude. Our model provides a useful tool for studying the various sensory processes possibly involved in dipterans’ flight stabilization abilities as well as the interactions between these processes.

## Introduction

Flying insects are subjected to a broad range of disturbances, for which fast, robust sensorimotor reflexes compensate. The flight stabilization performance of flies are even more impressive in view of the intrinsic aerodynamic instability of their flapping flight [1–4]. Compensating for this passive instability requires an active inner control of the wing kinematic in addition to an outer-loop system which responds to specific sensory cues (looming objects, odours and navigational cues). Discovering insects’ abilities to sense movement via optic flow crossing the compound eye or inertially via the halteres (for dipteran) is still of great interest. However, very few studies have focused so far on how flying insect sense their absolute body orientation in the three-dimensional space (attitude) with respect to a vertical reference. Oppositely, several studies have suggested that flies may lack the ability to perceive the vertical (via graviceptive cues) in order to stabilize their flight [5–7]. To address this point, we used an already designed free-fall procedure [7] with which insects can be briefly exposed to near-weightless conditions in a box lined with horizontal black and white stripes. The present study focused on the following questions:

1. whether an optic flow-based strategy may be involved in hoverflies’ speed control in closed-loop and prevent them efficiently from crashing after a free fall, and
2. whether this simple strategy may durably ensure flight stability.

The control of flight speed based on optic flow cues have been confirmed by ethological studies [8–10]. In the same time, in flying insects, as in helicopter, lift vector and body orientation are fixed in time and consequently flight speed and pitch orientation [1, 3, 11], and the idea that insects’ attitude may be stabilized on the basis of the optic flow has been tested successfully on a 2 degree-of-freedom flying robot [12]. A pitch rate control process has also been proposed previously to model the drosophila’s forward velocity during flight [13, 14]. Based on the existence of coupling between pitch control and optic flow regulation, we challenged the suitability of such closed-loop control compared with hoverflies subjected to an unsteady free fall situation.

It has been previously established that the fly’s auto-stabilizer involves several sensory modalities, which interact during flight. First, insect vision is based on two physical structures, compound eyes and ocelli. The fly’s photoreceptors feature a high temporal resolution giving them a great ability to detect fast motion based on contrast changes [6]. Optic flow measurement have shown that motion vision is involved in many visually guided tasks such as flight speed and altitude control, wall following, odometry and optomotor response [9]. Most of the optic flow processing is performed by compound eyes, and local contrast motion measurements are fused by lobula plate tangential cells (LPTC) responsible for detecting large field motion [15, 16]. In addition, it has been established that several groups of interneurons, including VSTCs (Vertical Sensitive Tangential Cells) [17] and HSTC (Horizontal Sensitive Tangential Cells) [18], process the various components of visual motion and in particular that they distinguish between the rotational and translational components of the optic flow with respect to the fly’s reference frame [19]. In addition, the ocelli, which are usually composed of three simple unfocused eyes forming a triangle at the top of the head [20], may be involved in the visuo-motor stabilization reflexes that maintain postural equilibrium by detecting the head’s rotational speed [21–25].

Dipteran also possess two minute dumbbell-shaped organs called halteres, which have evolved from hind-wings and beat simultaneously in anti-phase with wings. This active beating along with the campaniform sensilla provide flies with sensitivity to Coriolis forces and consequently to their own body’s angular speed [26–28]. The halteres enable the fly’s autopilot to respond to extremely abrupt changes in attitude with a latency as short as 5ms [29, 30]. In addition, insect’s hairs and antennae are sensitive to airflow during flight. Airflow sensing by the Johnston’s organs present in the antennae is known to be involved in flight speed regulation complementary to optic flow regulation [31, 32].

All in all, these sensorimotor units are mainly characterized in flies by their high temporal resolution and their low latency response [29]. Flies’ sensors are indeed highly tuned to detecting and quickly counteracting any change in their environment [6]. The combination of various sensory modalities with different bandwidth allows them to cover a wide range of dynamic perturbations.

In this study, an insect flight control model was developed, based simply on the closed-loop control of the pitch rate and the regulation of the horizontal component of the optic flow. In a first step, our model was devoid of any kind of absolute reference. The results obtained with this model simulating the fly’s response in unsteady free fall situations are compared with experimental data obtained on plummeting hoverflies in a box lined with horizontal black & white stripes. The model simulated data matched what occurred during the first few milliseconds of the insects flight, but the pitch and speed responses became highly unstable after around 0.4s. In the second step, the accuracy of the model’s predictions was greatly improved by including two additional feedback loops: one controlling the pitch rate on the basis of the absolute estimation of the pitch orientation and one controlling the lift and thrust forces on the basis of the vertical optic flow. The ability of the fly to measure its pitch orientation with respect to an absolute reference value is discussed in term of the existence of visually mediated responses such as the dorsal light response (DLR).

## Materials and methods

### Dynamic model for the closed-loop control of the pitch, speed and force in the freely falling fly

**Fig 1:**
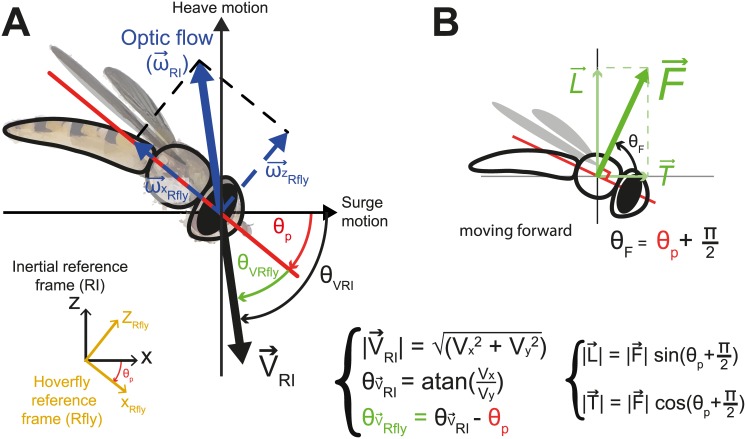
Main parameters of the model. (A) We take the theoretical optic flow vector (${\vec{\omega }}_{RI}$) to be the opposite of the 3-D speed vector (${\vec{V}}_{RI}$) experienced by the hoverfly. We calculated a horizontal ($\omega x_{R_{fly}}$) and a vertical ($\omega z_{R_{fly}}$) component of this theoretical optic flow vector in the hoverfly’s reference frame (*R*_*fly*_) depending on the estimated pitch orientation. (B) The force produced by the hoverflies’ flapping wings ($\vec{F}$) is assumed to be orthogonally oriented with respect to the body pitch orientation [11]. Moving forward is then achieved by pitching down from the head and moving backward, or braking, by pitching up from the head. Lift force ($\vec{L}$) corresponds to the vertical component of $\vec{F}$ in the inertial reference frame and thrust force ($\vec{T}$) to the horizontal component. As depicted in [35], we assumed a pure active control of the pitch torque which is seen to occur during a fraction of the wingstroke, about half of a wing beat period (i.e., about 2ms for an hoverfly, see [3]).

**Fig 2:**
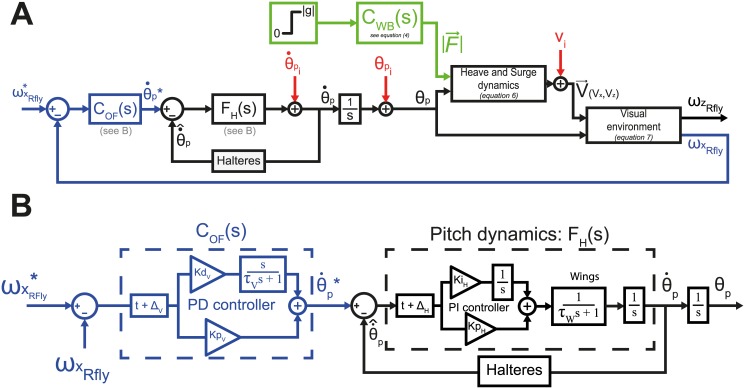
Model block diagrams. (A) Control of the pitch rate (${\dot{\theta }}_{p}$) in response to horizontal optic flow component (*ωx*_*Rfly*_). The measured *ωx*_*Rfly*_ is compared with an input reference set-point, $\omega x_{Rfly}^{*}$, and the error (*ϵ*_*ω*_) is then sent to a Proportional-Derivative controller, which delivers a reference pitch rate, ${\dot{\theta }}_{p}^{*}$. We then used a second loop mimicking the halteres, to measure and adjust the pitch rate, ${\dot{\theta }}_{p}$, to this set-point. The pitch *θ*_*p*_ obtained by integration (by definition) is then used to calculate the orientation of the thrust and the speed vector in the inertial reference frame (see B). The norm of the force produced by flapping wings is assumed to correspond to a second order transfer function with a zero based on the data. The two components of the optic flow vector in the fly reference frame, *ωx*_*Rfly*_ and *ωz*_*Rfly*_, are then calculated geometrically (see B). *ωx*_*Rfly*_ is used to close the pitch control loop. (B) Details of the pitch rate control based on horizontal optic flow component.

In a previous study on flight stabilization in plummeting hoverflies [7], we established that the flies’ crash avoidance performance depended more on visuo-motor reflexes than on gravity perception. In order to understand those reflexes more deeply, we modeled a fly’s pitch rate control system based on optic flow cues (see Figs 1 and 2) and compared the results obtained during model simulations with experimental free falling hoverflies. First, we focused on the pitch because we observed that during the period elapsing between the onset of the fall and wingbeat initiation, flies pitched down smoothly, probably because of the pin glued onto their thorax. Therefore, pitch was taken to be the main state to be controlled by the fly’s stabilizer to avoid crash. Secondly, since gravity cues do not seem to be involved in insect flight control [6, 7, 33], we assumed that fly’s flight control does not rely on any absolute vertical reference of the environment but that it is based rather on visual and inertial motion perception and compensation. We therefore based our model on previous studies on insects’ flight behaviour providing clear-cut evidence that optic flow-based control are involved during several tasks (for a review see [9]). We considered here that the forward speed was controlled by pitching-down from the nose the body and then orienting the force vector produced by flapping wings [34], as occurred in the case of the helicopter analogy [11]. The pitch rate is set so as to keep the forward optic flow constant, as found to occur in bees traveling in a textured corridor [10].

#### Closed-loop control of the body’s angular pitch rate via the halteres

The estimation of the pitch rate $\hat{{\dot{\theta }}_{p}}$ was assumed to be carried out by the halteres, in order to control in closed-loop the body’s rotational speed through a proportional-integral controller (*PI*_*H*_), as previously proposed for fruitflies [35]:

$$\begin{array}{c} \begin{array}{ccc} {\dot{\theta }}_{p} & = & PI_{H}*\left({\dot{\theta }}_{p}^{*}-\hat{{\dot{\theta }}_{p}}\right)\\ \text{where}\;PI_{H} & = & Ki_{H}*\frac{1}{s}+Kp_{H} \end{array} \end{array}$$ (1)

with ${\dot{\theta }}_{p}^{*}$ the set point, $\hat{{\dot{\theta }}_{p}}$ the estimation of body’s rotational speed, *Kp* = 7 and *Ki* = 0.3 (see [35]) and *s* the Laplace variable ($\frac{1}{s}$ is a pure integrator).

In this study, we modelled the angular speed control of the body pitch with a closed-loop involving the halteres combined with a simplified dynamic of the wing pitch control (a first order transfer function with a 0.002*s* time constant) based on the observation that corrective manoeuvres in flies occur in a single wing stroke time order corresponding to approximately 2*ms* [3].

#### Closed-loop control of the forward speed via the compound eyes

The pitch rate reference signal ${\dot{\theta }}_{p}^{*}$ is provided by a proportional-derivative controller (*C*_*OF*_(*s*)) based on the visual error between the measured forward optic flow ($\omega x_{R_{fly}}$) and a constant setpoint ($\omega x_{R_{fly}}^{*}=0.04cm.ms^{-}1$) arbitrary determined based on optic flow data (see Results section). A Proportional-Integral-Derivative (PID) controller was adopted in order to make the model as generic as possible and to keep in line with recent insect flight control studies [13, 14, 29, 35]. $\omega x_{R_{fly}}$ is considered in the fly’s reference frame as the longitudinal axis component of the overall optic flow vector, $\vec{\omega_{RI}}$, experienced by the insects (see Fig 1). This measurement is based on the existence of the HS cells in the lobula plate, which are sensitive to horizontal optic flow in the flies’ visual field [19]:

$$\begin{array}{c} \begin{array}{ccc} {\dot{\theta }}_{p}^{*} & = & PD_{V}*\left(\omega x_{R_{fly}}^{*}-\hat{\omega }x_{R_{fly}}\right)\\ PD_{V} & = & Kp_{V}+Kd_{V}*\frac{s}{\tau s+1}\text{;}\;\text{with}\;K_{p}\;\text{and}\;K_{d}\;\text{free}\;\text{parameters}\\ \hat{\omega }x_{R_{fly}} & = & |\vec{\omega_{RI}}|\;cos\left(\theta_{V_{R_{fly}}}\right) \end{array} \end{array}$$ (2)

#### Model of the flapping wing force dynamics

**Fig 3:**
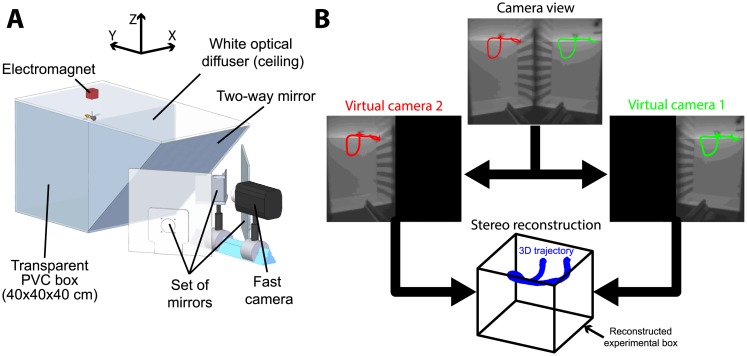
Setup and stereo reconstruction. (A) The setup consists of a 40x40x40cm^3^ box illuminated from above with a halogen light. Hoverflies were filmed in the box with a fast camera through a two-way mirror at a rate of 1600 frames per second in full resolution (1280 by 800 pixels). The mirror was tilted at an angle of 45° to make the hoverfly see a uniform white wall (i.e. the reflection of the white light-diffusing ceiling). A manual switch was used to trigger the camera and simultaneously turn off the power of the electromagnet, thus releasing the resting fly and causing it to fall. A set of mirrors was used to split the images, giving two different views of the experimental box. (B) Example of a split image obtained with the set of mirror with the two trajectories superimposed. The two trajectories were used to obtained a 3-D reconstruction with the MATLAB stereo vision toolbox.

To model the force dynamics with the transfer function F(s), we first estimated the horizontal and vertical acceleration of the hoverfly during the experiment by applying a double derivation of the measured fly´s position using stereo reconstruction methods (see Fig 3). The accuracy of the position measurement is given by eqs 9 and 10. The gravitational acceleration (*g* = −9.81) was subtracted from the vertical component and the overall acceleration produced by hoverflies was taken to be the vectorial summation of these two components *Axy* (the acceleration on the XY plane) and *Az* (the acceleration on the Z axis):

$$\begin{array}{c} \begin{array}{ccc} Axy & = & \sqrt{Ax^{2}+Ay^{2}}\\ |\vec{F}| & = & \sqrt{Axy^{2}+{\left(Az-g\right)}^{2}} \end{array} \end{array}$$ (3)

The data resulting from the double derivative procedure was filtered using a Savitzky-Golay filter of order 1 and with a frame length of 11. The force produced by the fly, $|\vec{F}|$, due to the wingbeat corresponds to the response of a second order low-pass filter with time constants *τ*_1_ = 0.01*ms* and *τ*_2_ = 0.1*ms* and a zero, *τ*_*z*_ = 0.24, to a step (0 to −*g*, see Fig 2A) which determines the transfer function, noted *C*_*WB*_(*s*) used to model the transient force production:

$$\begin{array}{c} C_{WB}\left(s\right)=\frac{F(s)}{U(s)}=\frac{0.24s+1.8955}{0.01s^{2}+0.1s+1};\;\;\text{with}\;\text{U(s)}\;\text{a}\;\text{step}\;\text{ranging}\;\text{from}\;\text{0}\;\text{to}\;\text{|g|.} \end{array}$$ (4)

The average lift vector was assumed to have a fixed position and orientation with respect to the body [1, 3, 11]. In addition, since hoverflies are known to hover keeping their body horizontal [4], an orthogonal relationship was assumed to exist between body and flapping wings’ force vector orientations. The orientation of the lift and thrust acceleration vectors are therefore calculated on the basis of both the simulated body pitch (*θ*_*p*_) and the absolute force ($|\vec{F}|$) values as follows:

$$\begin{array}{c} \begin{array}{ccc} L & = & sin\left(\theta_{p}+\frac{\pi }{2}\right)*\left|\vec{F}\right|\\ T & = & cos\left(\theta_{p}+\frac{\pi }{2}\right)*\left|\vec{F}\right| \end{array} \end{array}$$ (5)

The measured horizontal and vertical speed of the virtual insect are then calculated by integrating the forces L and T, and the x and z position by integrating the horizontal and vertical speeds as follows:

$$\{\begin{array}{lll} V_{z} & = & \int \left(g+\left|\vec{F}\right|sin\left(\theta_{F}\right)\right)dt\\ V_{x} & = & \int \left(\left|\vec{F}\right|cos\left(\theta_{F}\right)\right)dt \end{array}\;\{\begin{array}{lll} Z & = & \int \left(V_{z}\right)dt\\ X & = & \int \left(V_{x}\right)dt \end{array}$$ (6)

The the optic flow used to control the pitch rate was then calculated on the basis of the horizontal and vertical speed components and the fly´s pitch orientation:

$$\{\begin{array}{lll} \left|\vec{\omega_{RI}}\right| & = & -\frac{\left|V_{RI}\right|}{d_{wall}}\\ \theta_{VRfly} & = & \theta_{VRI}-\theta_{p} \end{array}\;\{\begin{array}{lll} \omega z_{Rfly} & = & |\vec{\omega_{RI}}|sin\left(\theta_{VRfly}\right)\\ \omega x_{Rfly} & = & |\vec{\omega_{RI}}|cos\left(\theta_{VRfly}\right) \end{array}$$ (7)

The body drag was neglected in our model as we observed in a previous study (supplementary material, [7]) that the range of flight speed experienced during free fall are always in a linear range.

#### Model for passive body tilting during the free fall

In addition to the sensorimotor loops at work during flight stabilization after the free fall situation, we added a passive component standing for the physical dynamics of the insects’ body during the actual free fall phase. The inertia of the fly´s body was not measured explicitly but included in the closed-loop dynamics of the pitch dynamics denoted *F*_*H*_(*s*) (see Fig 2B). This part of the model accounts for the passive torque that makes the fly pitching down before initiating the wingbeats. As shown in S2 Fig (supporting information), to estimate the passive pitch torque occurring before wingbeat initiation, we fitted a fifth-order transfer function, noted *T*_*passive*_(*s*), directly to the averaged free fall pitch data of all the experimental essays (before wingbeat initiation):

$$\begin{array}{c} T_{passive}\left(s\right)=\frac{\theta_{passive}}{U(s)}=\frac{0.001s^{2}+30s}{s^{2}\left(0.0011s^{3}+0.0005s^{2}+0.01s+1\right)} \end{array}$$ (8)

with U(s) a step ranging from 0 to $-\frac{pi}{2}$ and *θ*_*passive*_ the passive component of the body pitch.

The wingbeats in the model, which was triggered at random time ranging from 75ms to 150ms after the start of the fall, defined the initial state values, *Z*_*i*_ (initial vertical position), *Vz*_*i*_ (initial vertical speed), *θ*_*pi*_ (initial pitch orientation) and ${\dot{\theta }}_{pi}$ (initial pitch rate). The two control loops based on the optic flow are then activated to compensate for the initial pitch disturbance.

A complete block diagram representation of the model is given in Fig 2. All the simulations were conducted with MATLAB-Simulink with a sampling period of 1/1600s (0.625ms), corresponding to the camera´ frame rate. In the simulations, hoverflies were assumed to move in a straight line equidistantly (20cm) from the two walls of an infinitely long corridor with a textured wall (see supporting information, S1 Fig), thus the optic flow vector was taken to be the opposite of the insect’s speed vector divided by the constant distance from the wall equal to 20cm (half of the box width).

### Experiments on hoverflies

In order to parametrize the gains in the PD controller in charge of the visual optic flow process in the model (see Fig 2C), we conducted a series of experiments with hoverflies.

#### Animals

Hoverfly pupae (*Episyrphus balteatus*) were purchased (Katz Biotech AG, Baruth, Germany) and reared until hatching in a cage measuring 53x29x29cm^3^, which was subjected to a 12h light/12h dark cycle at a temperature of 25 ± 2.5°C. Newly hatched adults had *ad libitum* access to a pollen/sugar mixture and water, as well as to real flowers to stimulate flying behavior. A piece of entomological pin approximately 5 mm in length was glued to the dorsal part of the animals´ thorax, perpendicularly to their longitudinal axis (Fig 3A): the pin (≈ 5*mg*) weighed approximately 15% of the hoverfly’s mass (≈ 35*mg*). The animals´ flight and hovering abilities were then checked in the breeding cages. In the subsequent experiments, 14 hoverflies were tested (9 males and 5 females). Animals were aged from 3 to 21 days during the experiments.

#### Experimental procedure

In the present study, hoverflies were subjected to free fall conditions in an upgraded version of the setup previously presented in [7]. An electromagnet (TEAC RL-1615) was used to suspend the insects with their legs dangling from the ceiling of a 40x40x40cm^3^ box (see Fig 3A). The box was covered with a white diffuser (PMMA WH02, 3 mm thickness) and illuminated from above by a halogen light (Kaiser Studiolight H). Hoverflies were filmed through a two-way mirror with a fast camera (Phantom Miro M110) at a rate of 1600 frames per second at full resolution (1280 by 800 pixels). Four mirrors were used to split the camera’s field of view (FOV) into two sub FOVs with half resolution (640 by 800 pixels), giving us a stereo vision of the scene from which the flies’ 3-D trajectories could be reconstructed (see Fig 3B). Flies were then released to make them fall by switching off the magnetic field. The flies experienced near-weightlessness for a short period (less than 290ms maximally) before triggering their wingbeats.

A total number of 57 falls were conducted in the 40cm-high box, two sides of which were lined with horizontal black and white stripes 2.8cm wide, giving a spatial period of 0.06*c*/*deg* at a distance of 20cm. Three consecutive falls were conducted during each run; each individual can be subjected to several runs on different days. We always checked whether the hoverflies could fly with their glued pin in the breeding cages before and after each experiment to confirm that their flight abilities were not affected by the pin or if they crashed on the floor during previous experiment. Experimental data were taken into consideration from the initiation of the free fall to the instant when the hoverflies were able to either yield a positive vertical speed or fly for 300ms. After this maximal 300ms time window, we considered that the flight control might be based on other strategies rather than that consisting of maintaining a constant forward optic flow in a 40^3^*cm*^3^ box.

#### Image processing

The horizontal and vertical 2-D positions of the hoverflies image centroid moving over a uniform background were recorded in each split image using a MATLAB custom-made image processing program, and a 3-D trajectory was obtained using the MATLAB stereo vision toolbox. The stereo configuration produced by the set of mirrors was estimated with the MATLAB stereo vision toolbox to reduce construction errors.

To estimate the pitch orientation, head, thorax and abdomen positions were estimated in the two split images and the orientation of the longitudinal axis was reconstructed in 3-D. Pitch angles were assumed to range from $\frac{pi}{2}$ (head upward) to $-\frac{pi}{2}$ (head downward). We calculated a theoretical estimate of the optic flow vector experienced by hoverflies as the opposite of the instant speed vector divided by the distances to the lateral walls (ranging from 10 to 20cm approximately in the collected data), giving two vectors with the same orientation but two different norms. To simplify the estimation we only kept the vector with the maximal norm, produced by the nearest wall.

To measure the precision of our image processing, and especially that of our body pitch orientation estimation, the first method used consisted in assessing the resolving power of our stereo construction theoretically. The minimal measurable distance between two points (Δ*d*) in a standard dual camera could be calculated as follows [36]:

$$\begin{array}{c} \Delta d=\frac{d^{2}\;SW}{BL\;IW\;FL} \end{array}$$ (9)

where SW is the sensor width, BL is the distance between the two cameras, IW is the image width in pixels, FL is the focal length and d the distance to the object/point of interest. From the calibration of the stereo construction done with the MATLAB stereo toolbox we obtained *BL* = 333.5*mm*, *FL* = 30*mm* and *d*_*m*_ *ax* = 1531*mm* and *d*_*m*_ *in* = 1131*mm*, corresponding to the maximum and the minimum distances of the experimental box from the virtual cameras (front and back walls). The horizontal and vertical physical sensor sizes of the camera used during the experiments were 25.6*mm* and 16*mm*, respectively, and the horizontal and vertical image sizes were 1280 and 800 pixels, respectively. Δ*d* therefore ranged between 2.56*mm* and 4.68*mm*, which means that various points distants from each other by ≈ 0.5*cm* could be distinguished. This validate the estimation of orientation by the detection of the head and the tail of hoverflies with a body length ranging between approximately 1*cm* and 1.5*cm*. This resolution made it possible to calculate in 3d space the Quantization Positional Uncertainty (*QPU*_*rms*_, see [36]) as follows:

$$\begin{array}{c} QPU_{rms}=\frac{1}{\sqrt{12}}\;\sqrt{\Delta d^{2}}=\left[0.46;\;0.62\right]mm \end{array}$$ (10)

However, in addition to the uncertainty of our stereo reconstruction, our body pitch orientation estimates depend strongly on how precisely head and tail extremities of flies could be detected. In addition to the theoretical calculation of uncertainty, a calibration procedure was conducted to estimate the angular errors in the pitch orientation estimates (see S5 Fig). The errors associated with our procedure to estimate pitch orientation amount to approximately ±5° (0.08*radians*) but we note that the hoverflies´ yaw orientations may affect this estimation. The main problem with this detection method is that when flies are facing the camera, so they can be considered as a filled circle, it makes the recognition of head and tail almost impossible or at least unreliable. All the trials in which flies were facing camera during their fall were therefore eliminated from the analysis. However, for the present purposes, this error seems to have been sufficiently low since our data acquisition frequency (1600 Hz) allowed us to filter out any occasional erroneous data.

### Estimating the parameters

The parameters of the visual Proportional-Derivative controller (*PD*_*V*_), *K*_*p*_ and *K*_*d*_, were estimated directly from experimental data. We first selected only the trials in which flies triggered their wingbeats in less than 150ms after the onset of the fall and were able to compensate for the fall by reaching a positive vertical speed (i.e., a lift force superior to their weight), amounting 44 experimental trials. A simulated falls was then achieved and compared with each of the selected falls as described above with several combinations of *K*_*p*_, ranging from 0 to 20, and *K*_*d*_, ranging from 0 to 2. A likelihood estimation (MLE) map was obtained for each fall, giving 44 maps in all, from which we extracted the average map shown in S2A Fig.

## Results

**Fig 4:**
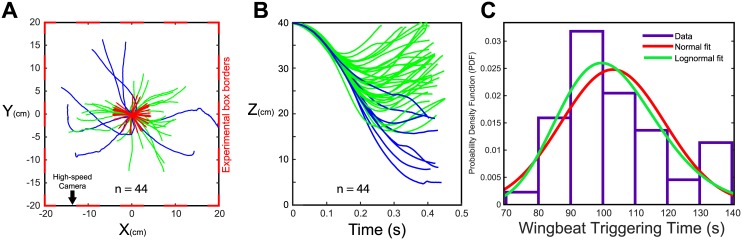
Measured 3D trajectories and wingbeat triggering times. (A) Hoverflies’ XY trajectories in all the trials in which flight was initiated before 150ms. Trajectories were stopped at the moment were animals overpass 0*cm*.*s*^−1^ vertical speed. Green lines correspond to stabilized flights, blue one to non stabilized flights. Red arrows indicate the direction between the start and the end of stabilized flight. Red dotted lines give the outlines of the experimental box. The high-speed camera is positioned approximately in coordinate (0,-100). The values on the X-axis are width coordinates and those on the Y-axis are depth coordinate. (B) Height of the hoverflies’ flight during the trials in which flight was initiated before 150ms. Green lines correspond to stabilized flights, blue lines to non stabilized flights. (C) Histogram of the times to initiate wingbeat after the onset of the fall observed during the experiments. Distribution has been fitted with both normal (red) and lognormal (green) rules distribution giving mean Δ_*WB*_ respectively equal to 103.121*ms* and 103.11*ms*.

As shown in Fig 4A (top view), in the box lined up with stripes only on the lateral walls (*X* = −20/20), the hoverflies did not seem to express any kind of preference for a specific wall. As expected from our previous study, it can be seen from Fig 4B that no crash occured in presence of visual cues (horizontal periodic stripes) and that most of the trajectories ended with a rising flight, which confirm the ability of hoverflies to control their flight in the free fall tests. The initiation times of the wingbeats, around 100ms in average (see Fig 4C), are also coherent with our previous findings [7]. In this study, we selected only trials featuring a time to wingbeat triggering inferior to 150ms to keep a sufficient margin from the 200ms time limit, after which it is impossible for the fly to stop its fall and avoid crash onto the ground [7].

**Fig 5:**
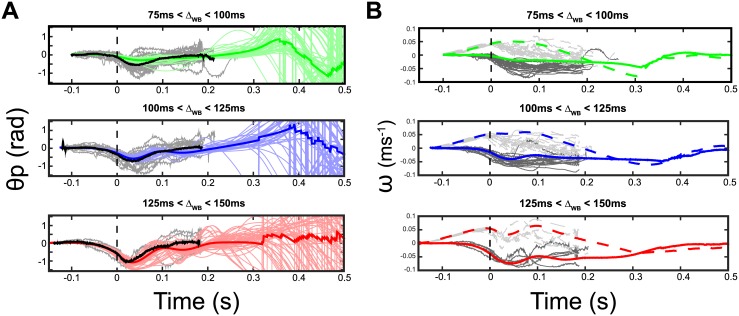
Model’s responses to a free fall (1). (A) Averaged simulated pitch orientation versus time in the 3 initiation time groups: 75 to 100ms (green line, top panel), 100 to 125ms (blue line, middle panel) and 125 to 150ms (red line, bottom panel). Gray lines are the experimental individual timelines. All the data were synchronized with the reference wingbeat triggering time: *t*_*WB*_ = 0. (B) Averaged simulated optic flows, $\omega x_{R_{fly}}$ (solid line) and $\omega z_{R_{fly}}$ (dashed line), versus time in the 3 initiation time group: 75 to 100ms (green line, top panel), 100 to 125ms (blue line, middle panel) and 125 to 150ms (red line, bottom panel). Gray lines show experimental data of $\omega x_{R_{fly}}$ (solid line, dark gray) and $\omega z_{R_{fly}}$ (dashed line, light gray).

Fig 5A (dark lines) shows the time course of the mean pitch orientation around the onset of the flies’ wingbeats. Hoverflies pitched down (i.e. head downward) when falling freely, but soon after initiating their wingbeats, they were able to compensate for the misalignment of their body tilt with respect to the horizontal within 150ms. Despite the existence of significant differences in pitch orientation at flight initiation, no difference were observed in terms of the final pitch orientation or correction times between late initiation (125–150*ms*), medium initiation (100–125*ms*) or early initiation (75–100*ms*), which shows the robustness of the reflex response involved.

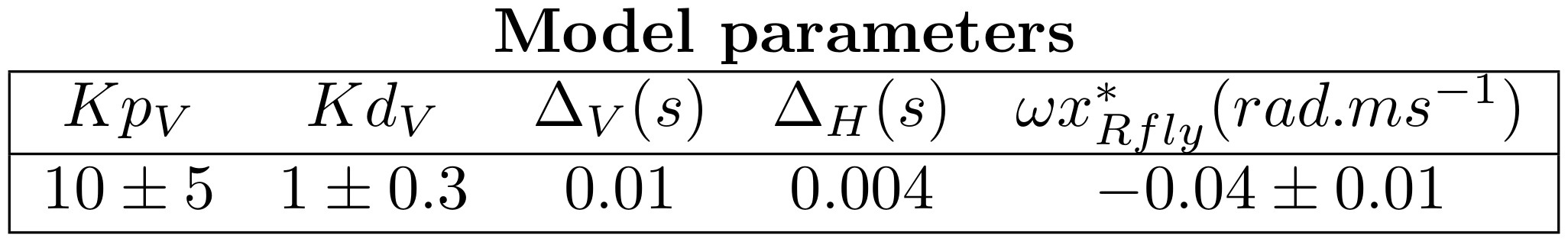

In Fig 5B (dark lines), the mean theoretical optic flow was calculated versus time during free fall and flight recovery phases. The vertical component of *ω*, *ωz*_*Rfly*_, increased to around 0.04*rad*.*s*^−1^ (for the latest initiation group) during the actual free fall and decreased quickly to zero after initiation of the wingbeats, whereas the horizontal component *ωx*_*RFly*_ decreased before the wingbeats was triggered and continue to decreased slightly after the initiation of the wingbeats and reached a mean steady state value of about −0.04*rad*.*s*^−1^ regardless of the initial conditions. This result supports the idea that hoverflies may control the optic flow in closed-loop so as to keep it constant during flight.

The results of the parameters identification, from which the parameters used during the simulations were selected, are presented in supporting information (S2 Fig). Fig 5 shows the results of 150 simulated free falls into the virtual 40cm width corridor and the parameters used.

The initial values used in simulation were determined by randomly setting a wingbeat triggering time ranging between 75 and 150ms to fit the data range (Fig 4C). The initial state of the system (i.e. the wingbeat triggering state), *θ*_*P*_*i*, ${\dot{\theta }}_{P}i$, *Zi* and *V*_*Z*_*i*, was obtained by simulating a free fall without any friction and adding a passive rotation of the body to the pitch dynamics before the onset of wingbeat triggering which was modeled by a third order transfer function (see supplementary materials, S2 Fig). The model accounted successfully for the dynamics of pitch orientation and optic flow observed experimentally during the 0.2s after the wingbeats initiation (see Fig 5).

**Fig 6:**
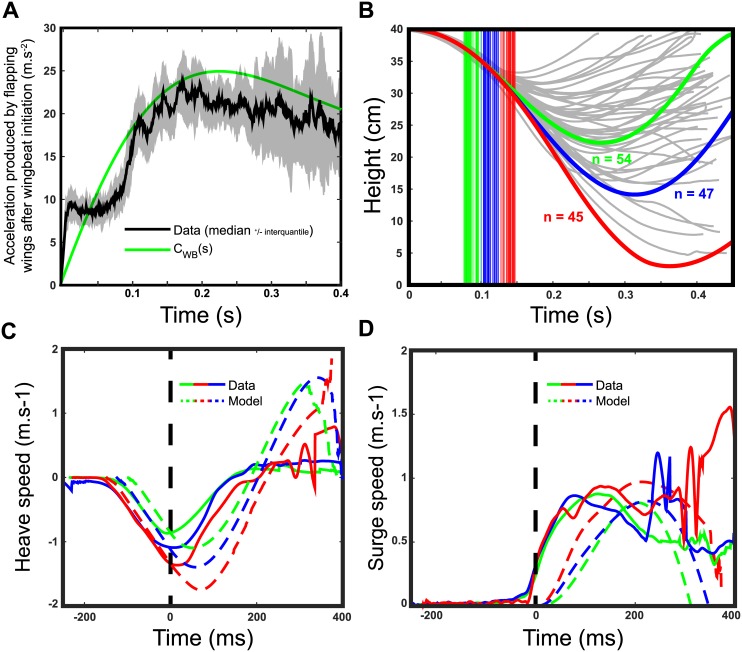
Model simulations in free fall (2). (A) Simulated acceleration produced by the flapping wings with time (green line) and median from thrust data ± interquantile deviation (dark lines). Time zero corresponds to wingbeat initiation. (B) Averaged simulated flight height versus time corresponding to wingbeat initiation time ranging from 75 to 100ms (green lines), 100 to 125ms (blue lines) and 125 to 150ms (red lines). Vertical bars show the wingbeat triggering times with the same color code. Grey shadowed lines are the 44 experimental time courses of the flight height. (C) Averaged heave speeds observed experimentally (solid lines) and in the simulation (dotted lines) in the three wingbeat initiation group: 75 to 100ms (green lines), 100 to 125ms (blue lines) and 125 to 150ms (red lines). (D) Averaged surge speeds observed experimentally (solid lines) and in the simulation (dotted lines) in the three wingbeat initiation group: 75 to 100ms (green lines), 100 to 125ms (blue lines) and 125 to 150ms (red lines).

Fig 6A shows the time course of the mean acceleration produced by the hoverflies, estimated from experimental data after subtracting gravity acceleration. After wingbeat initiation, the acceleration increased immediately to a value around 10*m*.*s*^−2^, which is equal to gravity acceleration absolute, during about 0.1*s*. After this initial phase, the acceleration increased within approximately 0.1*s* to a value of 25–30*m*.*s*^−2^, representing 2.5-3 times the absolute value of gravity acceleration, followed by a slight descent phase to around 20*m*.*s*^−2^ at 0.4*s*. The force produced by flapping wings in the model was adjusted to these dynamics as shown in Fig 6A (green line). However, the acceleration estimated from 3-D trajectory data is really noisy and could result in some discrepancies between simulated and experimental data. As it can be seen from Fig 5B, the average Z position observed during the simulations shows that the model was able to counteract the fall but the values obtained did not completely match the experimental data on the hoverflies. Nor did the heave and surge speeds match experimental data: they rather showed the occurrence of instability after around 200ms (Fig 5C and 5D).

## Discussion

In this study, control theory was used to model the pitch stabilization process at work in hoverflies placed in free fall situation, using simple rules based on optic flow measurements previously described in navigational tasks context [9]. We proposed a model (Fig 2) based on a virtual fly falling within a textured corridor accounting for the fly’s pitch and speed during about 200ms after the onset of the insect’s wingbeats. As in previous studies [9, 10, 13, 14], we assumed that the pitch control and hence the lift and thrust force control processes rely on the closed-loop control of the pitch rate via the halteres combined with an OF-based feedback loop. In line with [35] and [13, 14], we implemented in our model a pitch rate feedback-loop mimicking the halteres via a proportional-integrator (PI) controller. Recent results have suggested that sensory cues delivered by the eyes, halteres and antennae may interact via specific actions and coupling arrangements [37–39]. The present model involves then two nested feedback-loops, one featuring fast dynamics thanks to the halteres and one featuring much slower dynamics due to the presence of a double integrator between the pitch rate and the speed of the fly (see Fig 2). The OF was defined as the ratio between the fly’s speed and its distance to the wall which was constant during the fall and did not vary conspicuously during the 0.2s analyzed during insects’ flights. The simulated OF measurements are therefore very similar to the airspeeds apart from a different scaling due to *d*_*wall*_. As shown in Fig 1 and described by equation of $\vec{T}$, the forward OF can be controlled directly by adjusting the fly’s speed *V*_*RI*_ and thus by controlling the pitch. As shown in Fig 5, a non-null forward OF component was observed during the fall due to a passive pitching of the fly.

We simplified the model of hoverflies´ flight dynamics by neglecting any coupling between the pitch control and the other two rotational axes (roll and yaw). There were two main reasons for focusing only on the hoverflies´ pitch attitude control:

- to minimize the complexity of the model´s control scheme. Our model does not account for the entire repertoire of the fly in terms of manoeuvrability such as bank turns. The main objective here was instead to assess the performances of a system limited to a translational optic flow-based strategy.
- to take account for the fact that fly´ pitch responses was the only rotational axis affected during the free fall phase, probably due to the additional load of the entomological pin glued to the insect´ thorax. In view of the relatively straight trajectories observed in the XY plane (see Fig 4A), we then considered that the strategy used by hoverflies to stabilize their flight would mainly be an active pitch control.

The main characteristic of the model (see Fig 2) presented here is the total absence of any kind of vertical reference for controlling the pitch in the closed-loop system. This idea was based on previous data showing the absence of graviception in dipteran’s flight control [7]. This model accounts for the fly’s transient response during a period of up to approximately 0.2s from the onset of the flapping flight. However, as shown in Fig 4B, most of the stabilizing manoeuvres in response to the free fall situation occurred within 0.2s. The ability of the optic flow model to counteract the fall without requiring any information about the insect’s absolute orientation confirms that optic flow regulation, in addition to navigation processes, may play a stabilizing role [12]. Indeed, a slight tilting of the body and hence of the lift quickly led to a involuntary translation in the environment that results in generating optic flow. Thus, actuating the wingbeats motor system to cancel the generated OF would lead to correcting the attitude. In particular, during an instable flight, the gravity acceleration would induces a permanent increase in the speed toward the ground and a simple strategy such as maintaining a constant forward optic flow will therefore intrinsically induces the pitch to decrease with respect to the horizontal and therefore a restabilization. Indeed, as shown in, the drag force experienced during free fall can be neglected, at least in the range of our experimental paradigm, reinforcing the detection of any heave acceleration by the mean of optic flow variation. In addition, previous studies [8–10] on speed regulation based on optic flow strategy validate the implementation of such feedback loops in flight control system. Future experiments would allow to better describe these sensorimotor regulation by using moving gratings on the walls of the box or a virtual reality setup [40, 41] for example.

**Fig 7:**
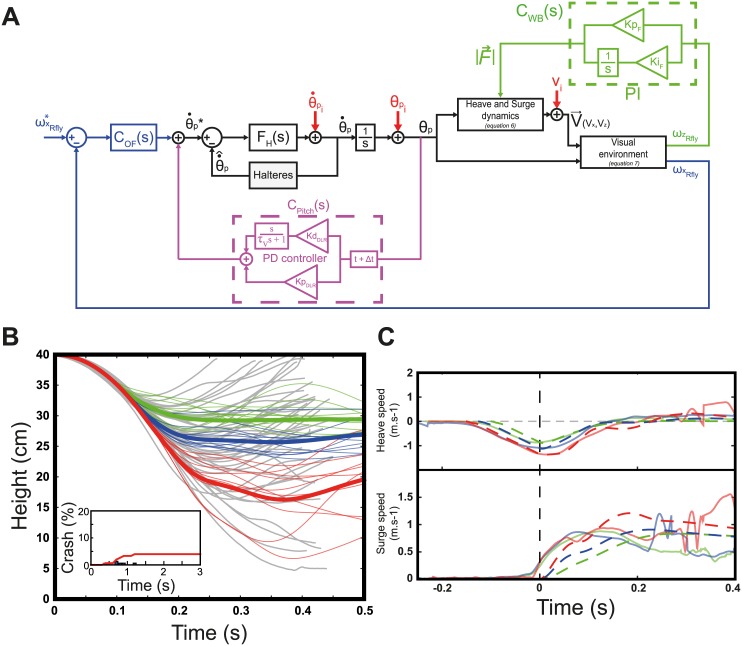
Block diagram of the improved model including a flapping wing force feedback control based on the vertical optic flow (green) and a pitch rate closed-loop control based on the absolute pitch measurement (purple). (A) Diagram of the model including a feed-back control loop based on *ω*_*ZRfly*_ accounting for the force produced by flapping wings and a feed-back control loop based on *θ*_*p*_ accounting for the control of ${\dot{\theta }}_{p}^{*}$. (B) Averaged (large solid line) and 10 samples (thin solid line) height measured during 150 simulations of free fall conducted with the new model. Model simulations results are presented in color (wingbeat triggering time of 75-100ms are shown in green, 100-125ms in blue and 125-150ms in red) and experimental data appears in grey. Inset shows the percentage of cumulated crash during 150 model simulations lasting 3 seconds. (C) Averaged heave (top panel) and surge speed (bottom panel) measured during the 150 simulations of free fall conducted with the new model. Model simulations are presented in dotted lines and experimental data in solid lines. Results are split in the three wingbeat initiation groups: 75-100ms are shown in green, 100-125ms in blue and 125-150ms in red.

**Fig 8:**
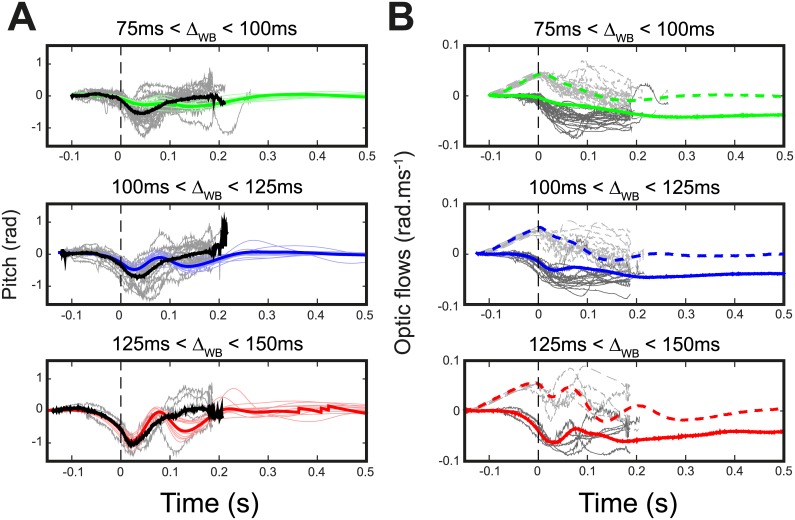
Experimental versus improved model responses in terms of pitch control dynamics. (A) Left panels give experimental (grey: individuals; dark: mean) and the simulated (color shaded: 10 individual samples; color plain: mean) pitch orientation versus time corresponding to the 3 groups of initiation time: 75-100ms are shown in green, 100-125ms in blue and 125-150ms in red. (B) Right panels represent the experimental (grey: individuals; dark: mean) and the simulated (shadowed: individuals; dark: mean) optic flows ($\omega z_{R_{fly}}$: dotted lines, $\omega x_{R_{fly}}$: solid lines) versus time corresponding to the 3 groups of initiation time: 75-100ms are shown in green, 100-125ms in blue and 125-150ms in red.

However, some instability in the model´s responses can be clearly seen to have occured after 300-400ms in Fig 6C. This means that a closed-loop pitch control system based only on the optic flow regulation does not suffice to maintain stable flight. Despite the presence of a fast closed-loop control of the pitch rate based on the halteres, a simple PD controller cannot be fast enough to stabilize a system featuring three integrators between the measured forward optic flow and the required pitch (see Fig 2 and eqs 6 and 7). Instead of increasing the complexity of the controller *C*_*OF*_(*s*), we decided to improve the model shown in Fig 2 by adding two biologically plausible feedback loops. First, we added a feedback loop controlling the lift based on the vertical flow $\omega_{z_{RI}}$ through a proportional-integrator controller. The integrator cancels the vertical optic flow while keeping a non-null steady lift force, thus simulating the altitude control process observed in dipteran [42, 43]. In addition, based on the existence of sensory mechanisms involved in the estimating pitch orientation such as the Dorsal Light Response and that based on an integration of the halteres’ and/or compound eyes’ signal, we added another pitch rate control loop including a Proportional-Derivative controller based on the absolute pitch orientation (Fig 7). Both additional PI and PD controllers has been set manually, gains are given in Fig 7 and all model parameters are summarized in S1 Table (supporting information). These two optic flow and pitch feedback loops combined made it possible to stabilize the simulated pitch and height in steady state (Fig 8). The ability of the improved model to stabilize the hoverflies´ attitude thanks to the addition of a closed-loop control of the pitch orientation suggests therefore a complementary control strategy involving pitch rate, pitch and optic flow measurements. With the model parameters presented here, a single pitch feedback loop would be too slow to stabilize the fly within 200ms as required by the 40*cm*-high box. Although, the ability of flying insects to estimate their absolute orientation (on the pitch and roll axis) still gives rise to some controversy, the model developed here would certainly provide a basis for studying these sensorimotor reflexes and the coupling that may exists between the sensory modalities involved. It is worth noting that [12] have established that the pitch of an aerial robot can be stabilized without any need for absolute reference value by regulating the dorsal and ventral OF of a 2 degree-of-freedom flying robot. Still our simulation gives controversial results in an unsteady situation such as free-fall recovering. Probably because in their study the rotor forces are adjusted by another control based on ventral optic flow in regard to experimental observation in bees [44]. In addition to visual motion and attitude perception, we can also assume that the fly could probably relies on others sensorimotor reflexes such as those based, for example, on the expansion of the OF [45, 46] that we did not challenge in this paper.

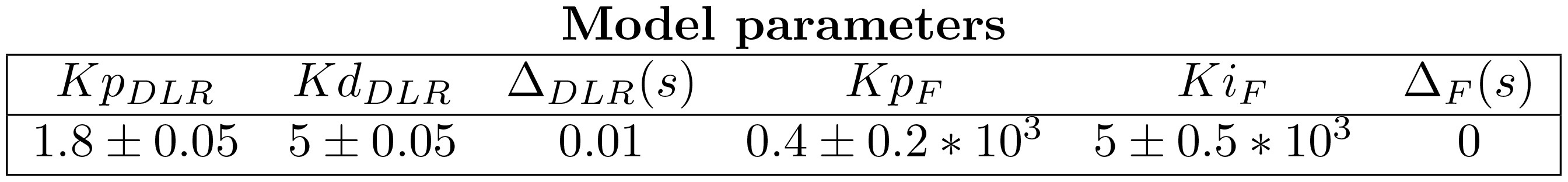

The hoverflies rely probably on specific sensory channels to estimate its absolute attitude with respect to its environment (i.e. a vertical reference) and to control their attitude as shown by the comparison made here between the two versions of the present model. Although our setup did not include any salient cues such an artificial horizon, the light from above may stimulate the DLR [22, 47, 48] which could help insects to estimate their attitude. However, a significant improvement in the hoverflies’ ability to stop falling before crashing have been previously observed when the insects were placed in a striped box rather than uniform white environment [7]. The reflex controlling the lift force orientation may not therefore relies solely on a pitch feedback based on the position of the brightest part of the visual field. A combination between optic flow based and DLR-based control may possibly be involved. However, the exact role of the DLR and its contribution to the visual-driven stabilization of insects’ flight is still an open question.

It is proposed in the future to investigate more closely how a pitch orientation could be estimated by hoverflies’ sensory system. An argument supporting the idea that pitch estimation is involved in the hoverflies’ response to free fall situations is the relative independence of the responses in regard to the initial conditions. In contrary, our model was found to be over-shooting in short initiation times (75-100ms) and under-shooting in long initiation times (125-150ms). To study the processes that can underlie the insects’ pitch orientation estimation, we can start with some hypotheses:

- the integral of body’s angular speed measured by halteres. This hypothesis has been supported in particular in a study by [29] on *drosophila* showing the reliability of a PI control for halteres compensating manoeuvers. Nonetheless, it is questionable whether halteres can measure body’s angular speed before wingbeat initiation because halteres are strongly wingbeat dependent.
- the rotational optic flow fields measured by the motion sensitive neurons may provide an estimation of the pitch rate and hence an estimation of the pitch also via its integral. Although, insects are known to be able to use the integral of translational optic flow for odometry purposes during navigation [9, 49]. Despite, halteres influence optomotor responses and visual motion detection [38] and the same dependence on wingbeats may inhibit any measurement before wingbeats.
- the ocelli response to a point light source (e.g. the sun) as a commonly accepted source for absolute (2-axis) body orientation reference [50].
- the dorsal light response (DLR) may have been triggered by our overhead lighting. There exist no evidence so far, however, that the DLR is actually involved in dipteran flight control apart from head stabilization process [51]. Only in locusts, the DLR has been shown to be involved in steering manoeuvers [48].

The accuracy of these four hypotheses still remains to be determined, along with the question as to whether any vertical information is really carried by one or more of these processes combined. The comparison with the optic flow strategy made here should help to determine how these various channels combined may serve to maintain a stable attitude during flight.

## Supporting information

S1 Fig Schematic representation of the simulations. In the simulations, the procedure was assumed to take place in an infinite corridor with textured walls. Numerically simulated hoverflies headed toward the corridor and their attitude varied only on the pitch degree-of-freedom. Since both yaw and roll are locked, they followed a straight line along the corridor. The optic flow therefore depended only on the insects’ surge speed.
(EPS)

S2 Fig Estimation of the model’s parameters. (A) Likelihood map averaged from the 44 parameters estimations. The histogram on the x- and y-axis give the distribution of the parameters selected with MLE procedure during individual estimations. (B) Pitch data before wingbeat initiation and step response of the transfer function *T*_*passive*_(*s*) modeling the dynamics of the passive pitching down from the nose during the free-fall. Averaged data and standard deviation are represented in dark line and grey area. The simulation of a fall is represented by the blue line.
(EPS)

S3 Fig **1—At the beginning of the film the head of the flies is dotted on each image from the two point of views.** 2—Three points are automatically tracked on each image of the flies: Head, thanks to the dot determined on the first image. Body, which is the centroid of the pixels’ mass. And tail, at the opposite extremity of the head. This step is repeated with 51 different binarization thresholds used for the body shape extraction (im2bw MATLAB function). 3—If head and tail are reversed during tracking, we can stop the tracking procedure, return to step 1 and start the automatic tracking again. 4—At the end of the tracking procedure, the 51x3x2D point clouds corresponding to each point of view are combined to construct 51x3x3D point clouds. From these clouds of points, pitch orientation of the body was determined at each time with 2 successive linear regression. First in the XY plane we estimate the yaw angle of the main body axis, the counter-rotation was applied to aligned this main axis with the X axis. Thus, in the ZX plane we can measure the main axis pitch orientation for each frame.
(EPS)

S4 Fig Procedure used to quantify the errors in the pitch orientation estimates. (A) Five 13mm long dummy hoverflies were printed in 3-D with a given orientation of their body in regard to their holding rod (ranging from 0° to 60°). They were suspended from a horizontal bar with two different yaw orientations, namely 0° and 90° and placed in 6 different positions on the horizontal plane of the experimental box (constant height: *Z* = 18*cm*). (B) A stereo system was used to track 3 body parts of the dummies, the head, the body center (the image centroid) and the tail from viewed from two different angles. (C) The 3-D reconstruction of the three parts of the dummy made it possible to reconstruct the 3-D body main axis and therefore to determine the dummy´ pitch orientation (see S3 Fig). A yaw orientation was estimated from the projection of the 3-D point cloud on the YX plane by performing a linear regression (*x* = *f*(*y*)) and the point cloud was corrected so as to align the main body axis with the Y axis. The pitch orientation was then estimated from the projection of the corrected 3-D point cloud in the ZY plane, again by performing a linear regression (*z* = *f*(*y*)).
(EPS)

S5 Fig Measurement of the pitch orientation estimation errors. (A) Bar histogram of the whole angular error in the case of the 6 positions in the experimental box and the two imposed yaw orientation of the 5 dummies (S4A Fig) shown in blue bars. Probability density function estimated with a t-location scale distribution in the case of the whole data (red line), the 90° yaw oriented dummies (green line) and the 0° yaw oriented dummies (cyan line). (B) Boxplot of the angular error in the case of all the positions and dummies pitch orientation, depending on the dummies´ yaw orientation. (C) Angular error of estimation versus the pitch and yaw orientation of the avatars. (D) Angular error of estimation versus the position of the dummies on the horizontal plane of the experimental box.
(EPS)

S1 Table Summary of the model parameters. (XLSX)
